# Comparative shotgun metagenomic data of the silkworm *Bombyx mori* gut microbiome

**DOI:** 10.1038/sdata.2018.285

**Published:** 2018-12-11

**Authors:** Bosheng Chen, Ting Yu, Sen Xie, Kaiqian Du, Xili Liang, Yahua Lan, Chao Sun, Xingmeng Lu, Yongqi Shao

**Affiliations:** 1 Institute of Sericulture and Apiculture, College of Animal Sciences, Zhejiang University, Hangzhou, China; 2 Analysis Centre of Agrobiology and Environmental Sciences, Zhejiang University, Hangzhou, China; 3 Key Laboratory for Molecular Animal Nutrition, Ministry of Education, Beijing, China

**Keywords:** Metagenomics, Entomology

## Abstract

Lepidoptera (butterflies and moths) is a major insect order including important pollinators and agricultural pests, however their microbiomes are little studied. Here, using next-generation sequencing (NGS)-based shotgun metagenomics, we characterize both the biodiversity and functional potential of gut microbiota of a lepidopteran model insect, the silkworm *Bombyx mori*. Two metagenomes, including the standard inbred strain *Dazao* (P50) and an improved hybrid strain *Qiufeng × Baiyu* (QB) widely used in commercial silk production, were generated, containing 45,505,084 and 69,127,002 raw reads, respectively. Taxonomic analysis revealed that a total of 663 bacterial species were identified in P50 silkworms, while 322 unique species in QB silkworms. Notably, *Enterobacter*, *Acinetobacter* and *Enterococcus* were dominated in both strains. The further functional annotation was performed by both BlastP and MG-RAST against various databases including Nr, COG, KEGG, CAZy and SignalP, which revealed >5 × 10^6^ protein-coding genes. These datasets not only provide first insights into all bacterial genes in silkworm guts, but also help to generate hypotheses for subsequently testing functional traits of gut microbiota in an important insect group.

## Background & Summary

Insects are the most diverse and largest class of animals on Earth, occupying in nearly all terrestrial ecological niches. Owing to this great diversity and the long-time coexistence, an amazing variety of symbiotic microorganisms have adapted specifically to insects as hosts, and participate in many relationships with the hosts^1–4^. In particular, the gut of most insects harbors a rich and complex microbial community with considerable metabolic activity^5,6^, which range from enhancing host energy metabolism to shaping immune system^7–11^. For example, various polysaccharide degrading bacteria were identified from herbivorous insect gut, which produce enzymes degrading otherwise host-indigestible plant component (e.g. cellulose, xylan)^12–16^. The native gut microbiota is also more and more recognized to play as an “extended immune system” for the host against harmful microbes^17^. Abundant lactic acid bacteria maintain in biofilms within honeybees (*Apis mellifera*) and work in a synergistic matter to inhibit pathogen proliferation in the gut by producing a mixture of antimicrobials^18^.

Although Lepidoptera, including butterflies and moths, is one of the largest insect orders and a primary group of phytophagous agricultural pests, little is known about the microbes associated with them^19,20^. Indeed, by using high-throughput sequencing techniques, recently several studies have reported abundant and diverse bacteria in lepidopteran guts^8,21–24^, but the functional significance of their gut microbiomes still remains undetermined. As a lepidopteran model organism and domesticated insect^25^, the silkworm *Bombyx mori* (Lepidoptera: Bombycidae) is important not only for basic research but also for providing raw materials to the textile and biotechnology industry^16,26–28^. Based on this model insect, the metagenomic analysis could form the basis for further research of lepidopteran microbiome.

**Figure 1:**
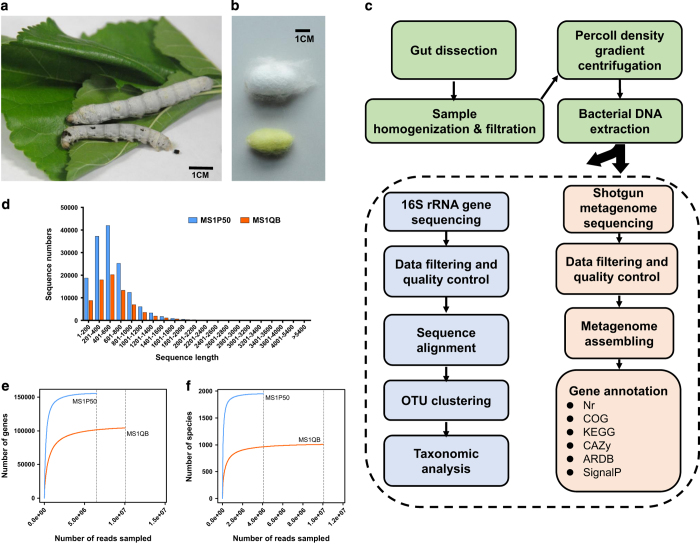
Silkworm (*Bombyx mori*) strains used for this study and overview of the experimental design. (**a**) The hybrid QB silkworm has a higher growth rate than P50. (**b**) Cocoon size and shape of P50 (yellow) and QB (white). (**c**) Workflow used to process silkworm gut samples to generate metagenomes. (**d**) Length distributions of filtered metagenome sequencing reads. (**e**) The functional gene rarefaction curve for each strain. (**f**) The taxonomical diversity rarefaction curve for each strain.

Here, using next-generation sequencing, we present shotgun gut metagenomes from two most common silkworm strains, namely *Dazao* (P50) and *Qiufeng × Baiyu* (QB). The inbred P50 silkworms are extensively used worldwide, as the standard strain for *B. mori* research; while the hybrid QB silkworms are widely used in local commercial silk production, which have a higher growth rate (Fig. 1a) and a larger cocoon size than P50 (Fig. 1b). Sample information was detailed in Table 1. As a herbivorous insect, the gut of silkworm is full filled with plant tissues, making it necessary to separate bacterial cells from the gut content to avoid plant DNA contamination. Thus, a filtration and density gradient centrifuge procedure was applied to enrich gut bacteria from the silkworm^13^. After bacterial DNA extraction, the metagenome was sequenced and analysed as the flowchart shown in Fig. 1c.

**Figure 2:**
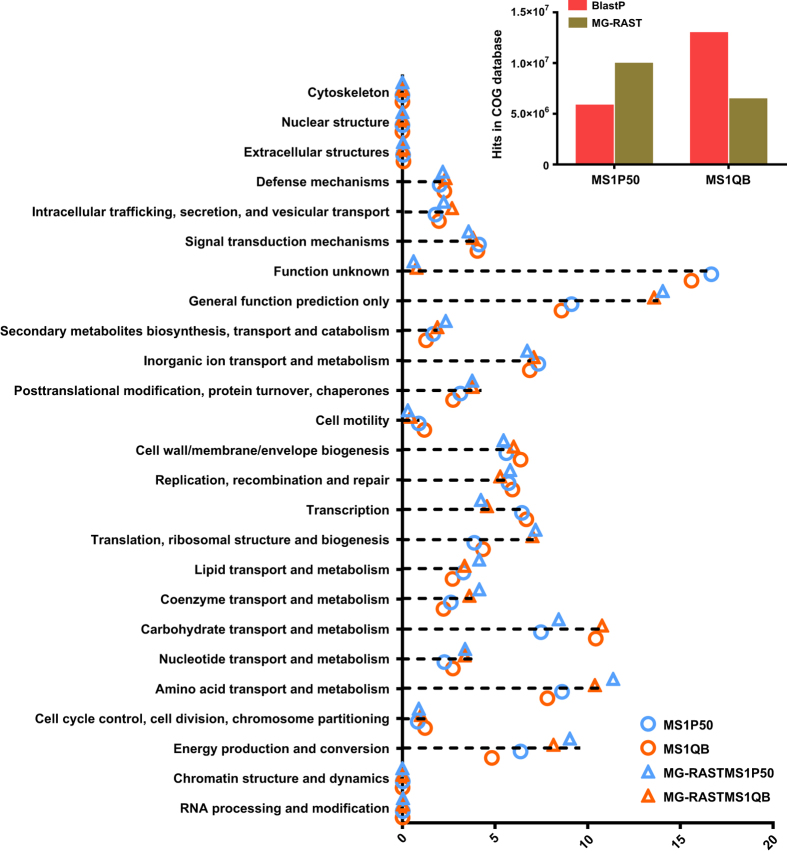
Composition of metabolism category in COG. BlastP (denoted with circle symbols) and MG-RAST (denoted with triangle symbols) methods are used respectively to query against COG database. Best hits of two strains are shown in the bar plot in the upper right panel.

**Figure 3:**
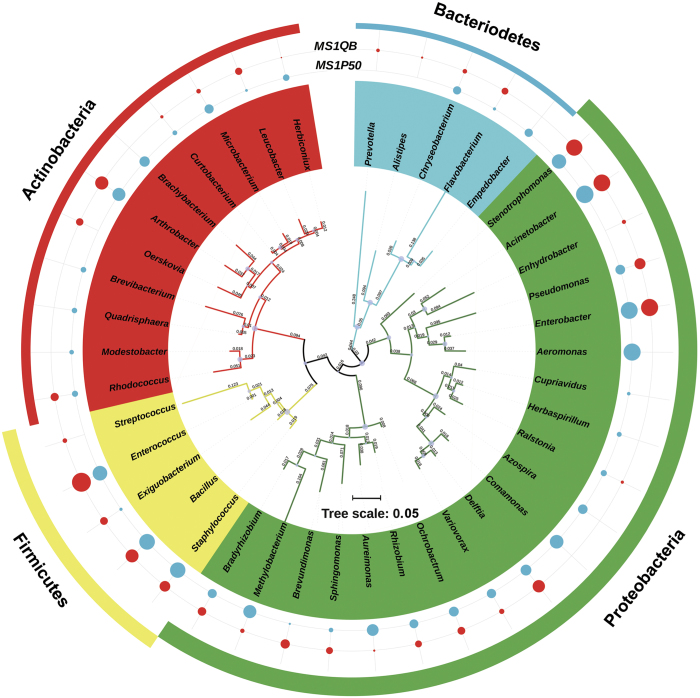
Species abundance and phylogenetic relationship of gut bacteria between P50 and QB. The phylogenetic tree is shown at the genus level, colored by the phylum. Bacterial abundance is indicated in outer ring with shape plot (P50, blue circle; QB, red circle). The size of circle represents sequence log10 reads per genus.

Shotgun sequencing produced 6.826 and 10.369 Giga base pairs (Gbp) of unassembled sequence data from P50 (MS1P50) and QB (MS1QB) samples (Table 2). In total, 44,047,886 and 67,718,490 sequences passed the quality control in the MS1P50 and MS1QB dataset, respectively. Read length distribution after filtering revealed most of sequences between 201–600 bp (Fig. 1d), and rarefaction curves tended towards saturation (Fig. 1e,f). The metagenomes were assembled separately into 91,037 and 44,201 scaffolds with 53.91 and 54.49% GC content in P50 and QB, respectively (Table 2). After ORF prediction, 148,685 ORFs from MS1P50 and 75,232 ORFs from MS1QB were identified. Table 3 summarizes functional gene annotation against various databases. By using both BlastP and MG-RAST protocols, the metabolism was found to be the major part of silkworm gut microbiome function (Fig. 2). From the Nr database output, 2,307,446 and 5,036,416 reads of P50 and QB were aligned to this category respectively, indicating that nutrient digestion and synthesis were most important aspects and microbial fermented products such as lactic acid, butyrate and vitamins^29–31^, could also be supplied to the host. Table 4 reveals that most reads ( >99%) identified from silkworm gut metagenome belong to the domain Bacteria. Taxonomic diversity was analysed not only with shotgun metagenomic data by different tools (BlastP against Nr database and MG-RAST against GenBank), but also by direct 16 S rRNA sequencing^32^, which showed the same tendency (Table 5). *Enterococcus*, *Acinetobacter*, *Bacillus* and *Enterobacter* are dominant species in both strains (Fig. 3) and have previously been found in silkworms^33^.

Altogether, the community structure and functional genes described here could be used for further exploring the potential relationship between the Lepidoptera host and commensal bacteria, thereby paving the way for developing novel strategies to promote silk production and enhancing studies on insect symbiosis.

## Methods

### Insect rearing and sample collection

Eggs of P50 and QB were provided by the Silkworm Quality Inspection and Quarantine Station, Department of Agriculture, Zhejiang Province, China. Silkworms were hatched in an incubator at 28 °C, 100% RH, and maintained in sealed plastic boxes (50 × 25 × 10 cm) with light-dark regime (16:8) at 25 °C and 70% RH. The 5^th^-instar larvae feeding with fresh mulberry leaves were used in this study. After 5 days feeding *ad libitum*, a hundred of P50 and QB silkworms were collected.

Gut dissection was performed as described previously^34^. Freeze-killed silkworms were washed with ddH_2_O for three times after surface-sterilization in 70% ethanol for 30 s. The larvae were dissected on ice in a clean bench. Considering that a large amount of bacterial DNA is needed for shotgun metagenomic sequencing and a risk for sample loss during purifying bacteria, dissected guts were pooled for DNA extraction and subsequent sequencing. Briefly, for each silkworm strain, 100 guts were homogenized with a hand-held homogenizer (PRO scientific, Monroe, USA). In order to avoid the plant and host tissue debris contamination, several steps of filtering were applied followed by a protocol specific for gut bacteria enrichment^35^. 100 mL 10 mM MgSO_4_ was then added to homogenized samples before being passed through 20 μm and 11 μm filters (Millipore, Bedford, USA). Each sample was centrifuged at 4000 rpm for 15 min. The pellet was resuspended with 200 μL ddH_2_O for further separation. 40 and 80% Percoll (GE Healthcare, Uppsala, Sweden) containing 10 mM MgSO_4_, 0.01% bovine serum albumen, 0.01% ficoll (Sangon, Shanghai, China), 0.05% polyethyleneglycol 6000 (Sangon, Shanghai, China), and 0.086% sucrose were used for the density gradient centrifugation. Each 7-mL centrifuge tube (Hitachi, Tokyo, Japan) was filled with 2.5 mL 80% Percoll on bottom layer, and 3.5 mL 40% Percoll on the top layer. Next, 1 mL of the sample was placed gently on the top of 40% gradient layer. The prepared tubes were centrifuged at 12,000 rpm, 4 °C for 10 min and bacterial cells were collected at the interface between the 40 and 80% Percoll solutions. Collected cells were washed by 1 mL of 10 mM MgSO_4_ for three times (4000 rpm, 5 min). The pelleted bacteria were used for DNA extraction.

### DNA extraction and shotgun metagenomic sequencing

To remove the host genetic contamination in samples, DNase I (Epicentre, Madison, USA) was added in and incubated at 37 °C for 30 min. After DNA digestion, 1 volume of 0.5 M EDTA (Sangon, Shanghai, China) was added and heated at 72 °C for 2 min to inactivate the DNase enzyme. Bacterial DNA was extracted from the washed cells by using the MasterPure™ Complete DNA and RNA Purification Kit (Epicentre, Madison, USA). The extracted DNA was quantified for the Illumina high-throughput sequencing (Illumina, San Diego, CA). 3 μg genomic DNA per sample was used for the sequencing library preparation. DNA samples were first fragmented into 300 bp fragments by the Covaris M220 shearing system (Covaris, Woburn, USA). Illumina TruSeq™ DNA Sample Prep Kit (Illumina, San Diego, CA) was then employed for the generation of 150 bp pair-end (PE) libraries following manufacturer’s recommendations. Illumina cBot Truseq PE Cluster Kit v3-cBot-HS (Illumina, San Diego, CA) was used for PCR reaction to enrich DNA fragments with ligated adapters on both ends. NGS sequencing was performed by Illumina Hiseq 2500 sequencing platform (Illumina, San Diego, CA), resulting two fastq files for each run.

### Metagenome sequence processing

Sequences with an average read length of 150 bp were introduced into SeqPrep (https://github.com/jstjohn/SeqPrep) to remove the adapter at 3′ ends of raw reads. Raw sequences containing 3 or more unknown nucleotides (‘N’) were trimmed by Sickle (https://github.com/najoshi/sickle) command “sickle pe” to obtain clean reads longer than 20 bp, and to ensure that filtered reads possessed quality threshold greater than 20. Clean reads were then assembled by SOAPdenovo^36^ at 39–47 *k*-mers. Scaffolds &lt; 500 bp were excluded. To construct a non-redundant gene set, CD-HIT^37^ was employed to cluster assembled reads at 90% coverage and 95% identity, using the longest read as the representative sequence. All high quality reads were aligned (95% identity) against non-redundant database using SOAPaligner (http://soap.genomics.org.cn/) to obtain the gene abundance in each sample.

### Metagenome annotation

After extraction of the non-redundant gene set, annotation was performed against Nr database by BlastP (v2.2.28+ ) at an e-value cutoff of 10^−10^, and the dataset was also processed by the web-based metagenomics RAST server (MG-RAST)^38–40^. Various databases (Table 3) were used for annotation^41^. KEGG^42^ and COG^43^ were employed too for the alignment of functional genes. To get the information about carbohydrate active enzymes and antibiotic resistant genes, sequences were compared in the CAZy^44^ and ARDB^45^ databases, respectively. Proteins transported through the secretory pathway, which contain signal peptides, were identified by SignalP programme^46^. The ratio of annotated genes were determined by read number of the hits to the non-redundant database. From the annotation results, contamination sequences of the host silkworm were removed. Rarefaction curve was generated by R package vegan, based on the output file of Nr annotation.

### 16S rRNA sequencing and taxonomic analysis

For amplicon sequencing library preparation, a 50 μL PCR reaction system containing 10 μL 5 × FastPfu reaction buffer, 2.5 U FastPfu Polymerase (Transgene, Beijing, China), 250 μM dNTPs, 200 nM of primers, 1 μL of DNA sample and DNA-free water was performed twice to link the sequencing adapter to the PCR products. The resulting fragments were pooled together equally and quantified by Quantifluor dsDNA system (Promega, Madison, USA). PE sequencing was performed on an Illumina MiSeq instrument (Illumina, San Diego, CA).

Raw PE reads were merged by FLASH software (v1.2.7), and trimmed with Trimmomatic (v0.36) (Q > 20, N bases < 1%). Clean reads were run through UCHIME (v7.1) to remove all chimeric sequences. UCLUST implemented in QIIME (v1.8.0)^47^ was used for OTU clustering at threshold 97%, then the representative sequences were selected by the pick_rep_set.py script in QIIME. Taxonomic classification was performed using RDP Classifier (v2.12) with a confidence cutoff of 0.8. Phylogenetic tree was used to exhibit the gut microbiota composition of silkworm^48^. The longest read of each bacteria genus was picked out to generate the tree (Maxium-likelihood tree, Tamura-Nei model and bootstrap 1000)^49^, and visualized using iTOL^50^. Unclassified OTUs were further identified with BLASTN^51^.

### Code availability

No custom code was used to generate or process these data. Software versions employed are as follows:

BlastP (v2.2.28 + )

FLASH (v1.2.7)

Trimmomatic (v0.36)

UCHIME (v7.1)

QIIME (v1.8.0)

RDP Classifier (v2.12)

iTOL (v3)

## Data Records

Raw data of shotgun metagenomic sequencing (fastq file) are available from the NCBI’s Sequence Read Archive (Data Citation 1 and Data Citation 2). Raw data of 16 S rRNA sequencing (fastq file) are available from the NCBI’s Sequence Read Archive (Data Citation 3 and Data Citation 4). All shotgun metagenomic sequencing data can be also found at the MG-RAST server (for the strain P50: http://www.mg-rast.org/mgmain.html?mgpage=overview&metagenome=mgm4767611.3; strain QB: http://www.mg-rast.org/mgmain.html?mgpage=overview&metagenome=mgm4754041.3). The annotation outputs generated in this work include the non-redundant gene set.fa file (Data Citation 5) and the Annotation_results.xlsx file produced through BlastP, MG-RAST, SignalP, and comparisons to the CAZy and ARDB databases (Data Citation 5). The outputs of taxonomic analyses include: (i) an OTU table BIOM file with taxonomic assignment generated from 16 S rRNA sequencing (Data Citation 5) (ii) bacterial abundance at the genus level (Data Citation 5) (iii) aligned sequences for generating the phylogenetic tree (Fig. 3) in Newick format (Data Citation 5), and (iv) taxonomic assignment based on all bacterial gene sequences identified in the shotgun assemblies (Data Citation 5).

## Technical Validation

Like other herbivorous caterpillars, the silkworm infests a large amount of plant tissues and its gut full filled with mulberry leaf materials. Therefore, direct DNA extraction from gut content for metagenome analysis commonly fails to capture sufficient bacterial sequences, being masked by overwhelmingly abundant plant DNA sequences, such as the chloroplast contamination. To overcome this limitation, we first filtered the homogenized gut tissue to remove most of plant particles from our sample. Then a Percoll-based density gradient centrifugation was performed to enrich gut bacteria accordingly^13^. Moreover, before DNA extraction, DNase was applied to remove host DNA contamination from bacterial cell suspension, finally this enzyme was inactivated by heating at 72 °C for 2 min.

For assessing shotgun metagenome data, we compared two universal protocols with the dataset, namely BlastP and MG-RAST^38,39^, both providing integrative analyses of sequences. Overall, the MG-RAST analysis agreed with BlastP analysis for both taxonomy and functional results (Fig. 2), indicating that there was no obvious technical bias for analyzing shotgun metagenomes in this study. Furthermore, for the 16 S rRNA gene classification, BLASTN was employed to identify the reads labelled “unclassified” at the genus level. The comparison of bacterial taxonomy data between 16 S rRNA sequencing (based on the RDP database) and shotgun metagenomic sequencing (based on Nr database) results verified the community structure.

## Additional information

**How to cite this article**: Chen, B. *et al*. Comparative shotgun metagenomic data of the silkworm *Bombyx mori* gut microbiome. *Sci. Data*. 5:180285 doi: 10.1038/sdata.2018.285 (2018).

**Publisher’s note**: Springer Nature remains neutral with regard to jurisdictional claims in published maps and institutional affiliations.

## Supplementary Material



## Figures and Tables

**Table 1:** Sample information in this study.

Sample	Biome	Feature	Material	Geographical location	GeoPosition	Protocol
MS1P50	Insect gut	Digestive tract environment	Gut tissue	Hangzhou of Zhejiang province, China	120.098057, 30.305965, 5 m	Shotgun Metagenome
MS1QB	Insect gut	Digestive tract environment	Gut tissue	Hangzhou of Zhejiang province, China	120.098057, 30.305965, 5 m	Shotgun Metagenome
16SSP50	Insect gut	Digestive tract environment	Gut tissue	Hangzhou of Zhejiang province, China	120.098057, 30.305965, 5 m	16S rRNA amplicon
16SSQB	Insect gut	Digestive tract environment	Gut tissue	Hangzhou of Zhejiang province, China	120.098057, 30.305965, 5 m	16S rRNA amplicon

**Table 2:** Metagenome sequencing statistics reported in this study.

Sample	MS1P50	MS1QB
**Sequence size (Gbp)**	6.826	10.369
**No. of reads**	45,505,084	69,127,002
**Sequence strategy**	Paired-end	Paired-end
**Library insert size**	500	500
**Average read length**	150	150
**No. of sequences removed by quality control procedures**	1,457,198	1,408,512
**No. of sequences that passed quality control procedures**	44,047,886	67,718,490
**Number of scaffolds ( >500 bps) after assembled**	91,037	44,201
**Total Bases in scaffolds > 500 bps**	93,065,111	49,571,885
**% of Sequences assembled**	93.50%	98%
**No. of singletons after assembly**	0	0
**N rate**	0%	0%
**Largest scaffold length (bp)**	18,177	52,980
**N50 scaffold length (bp)**	1,076	1,214
**N90 scaffold length (bp)**	564	578
**GC content**	53.91%	54.49%
**ORFs ( >100 bps)**	148,685	75,232
**Average ORF length (bp)**	546.7	576.3

**Table 3:** Annotation summary.

Databases	Numbers of reads annotated	
	MS1P50	MS1QB
**Nr**	6,474,494	14,568,868
**COG**	5,842,302	13,008,590
**KEGG**	3,333,526	7,823,300
**CAZy**	175,548	546,668
**ARDB**	10,040	27,756
**SignalP**	437,774	945,626

**Table 4:** Domain coverage of shotgun reads.

Domain	Shotgun reads	
	**MS1P50 (%)**	**MS1QB (%)**
**Archaea**	22 (0.00%)	440 (0.01%)
**Bacteria**	11,706,480 (99.94%)	4,055,040 (99.23%)
**Eukaryota**	5,858 (0.05%)	27,190 (0.67%)
**Viruses**	810 (0.01%)	3,784 (0.09%)
**No rank**	18 (0.00%)	2 (0.00%)

**Table 5:** Taxonomic composition (top 15) revealed by 16S rRNA sequencing and shotgun metagenomic sequencing.

Genus	16S rRNA sequencing (RDP database)		Shotgun metagenome sequencing (Nr database)	
	16SSP50 (%)	16SSQB (%)	MS1P50(%)	MS1QB (%)
***Enterococcus***	1,675 (5.0%)	14,576 (43.8%)	765,432 (18.7%)	7,412,022 (63.3%)
***Acinetobacter***	7,117 (21.3%)	4,895 (14.7%)	663,036 (16.2%)	340,064 (2.9%)
***Enterobacter***	5,295 (15.8%)	6,021 (18.1%)	198,836 (4.9%)	1,265,284 (10.8%)
***Aeromonas***	5,954 (17.8%)	0 (0%)	354,508 (8.7%)	150 (0.0013%)
***Stenotrophomonas***	1,491 (4.4%)	3,773 (11.3%)	69,468 (1.7%)	785,760 (6.7%)
***Bacillus***	2,910 (8.7%)	728 (2.2%)	225,798 (5.5%)	54,856 (0.47%)
***Staphylococcus***	2,473 (7.4%)	224 (0.67%)	56,150 (1.4%)	58,798 (0.50%)
**Planococcaceae_unclassified**	2,064 (6.2%)	111 (0.33%)	–	–
***Arthrobacter***	734 (2.2%)	793 (2.4%)	57,282 (1.4%)	11,268 (0.096%)
***Comamonas***	1,004 (3.0%)	0 (0%)	27,974 (0.68%)	8,644 (0.074%)
***Delftia***	242 (0.70%)	534 (1.6%)	100,894 (2.5%)	139,532 (1.2%)
***Methylobacterium***	751 (2.2%)	9 (0.027%)	12,394 (0.30%)	254 (0.0022%)
**Enterobacteriaceae_unclassified**	20 (0.05%)	606 (1.8%)	—	—
***Pseudomonas***	138 (0.41%)	269 (0.81%)	25,140 (0.62%)	122,492 (1.0%)
***Aureimonas***	389 (1.2%)	3 (0.0090%)	2,590 (0.063%)	6 (0.00%)
**Total reads**	33,473	33,254	4,086,456	11,713,188
